# Association of endothelial nitric oxide synthase (Glu298Asp) gene polymorphism with radial artery spasm during cardiac catheterization in Egyptians

**DOI:** 10.1007/s11033-023-08434-0

**Published:** 2023-05-23

**Authors:** Tarek A Abdelaziz, Randa H. Mohamed, Ashraf A Dwedar, Mohey Eldeen A Eldeeb, Abdelrahman A Abdelfattah, Sara F Saadawy

**Affiliations:** 1 Cardiology Department, Faculty of Medicine, Zagazig University, Zagazig, Egypt; 2 Medical Biochemistry Department, Faculty of Medicine, Zagazig University, Zagazig, Egypt

**Keywords:** Radial artery spasm, eNOS gene, Glu298Asp polymorphism

## Abstract

**Background:**

Nitric oxide (NO) exerts diverse effects on the cardiovascular system. Impairment of NO production plays a key role in cerebral and coronary artery spasm. We aimed to explore the predicting factors of radial artery spasm (RAS) and the association of eNOS gene polymorphism (Glu298Asp) with RAS during cardiac catheterization.

**Methods and results:**

200 patients underwent elective coronary angiography through a trans-radial approach. The subjects were genotyped to the Glu298Asp polymorphism (rs1799983) on the eNOS gene by polymerase chain reaction-restriction fragment length polymorphism (PCR-RFLP). Our results showed that the subjects with the TT genotype and T allele were significantly more likely to develop radial artery spasms (OR = 12.5, 4.6, P < 0.001 respectively). TT genotype of eNOS Glu298Asp polymorphism, number of punctures, size of the radial sheath, radial tortuosity, and right radial access are independent predictors of radial spasm.

**Conclusion:**

The eNOS (Glu298Asp) gene polymorphism is associated with RAS during cardiac catheterization in Egyptians. TT genotype of eNOS Glu298Asp polymorphism, number of punctures, size of the radial sheath, right radial access, and tortuosity are independent predictors of RAS during cardiac catheterization.

**Supplementary Information:**

The online version contains supplementary material available at 10.1007/s11033-023-08434-0.

## Introduction

A trans-radial approach (TRA) is being used worldwide in both emergency and elective procedures of cardiac catheterization and percutaneous coronary intervention (PCI). Several factors contribute to explaining this trend, including patient comfort, early mobilization, and a significant reduction in a hospital stays. when compared to the transfemoral approach, TRA reduced major bleeding and all-cause mortality [1, 2]. Radial artery spasm (RAS) is still an important limitation of TRA. Previous studies reported incidence of RAS was 14.7% [3]. Identification of RAS predictors, despite proposed vasodilators and different available sheaths and catheters, is an important issue in patient selection and avoidance of complications, including RAS [4]. Prevention of RAS is more effective than the treatment of spasm, which is why it is so important to know the predictive factors of this event [5].

The increased incidence of radial artery spasm is due to decreased nitric oxide release as well as a reduced endothelium-derived hyperpolarizing factor [6].

Nitric oxide (NO) is synthesized by the endothelial nitric oxide synthase (eNOS), which is a product of the (eNOS) gene. The Glu298Asp polymorphism in the exon-7 of the eNOS gene has been investigated to determine the relationship of polymorphism to atherosclerosis and coronary artery disease (CAD) [7, 8]. The eNOS (Glu298Asp) gene polymorphisms predispose the patients to arterial spasm [9], but no specific study had been done in correlation with RAS during cardiac catheterization.

Despite the widespread use of TRA, very few studies have focused on the association of risk factors with the occurrence of RAS in cardiac catheterization [10].

This study aims to explore the predicting factors of radial artery spasm and the association of eNOS (Glu298Asp) gene polymorphism with RAS during cardiac catheterization.

### Study patients

The present study included 200 Egyptian patients who underwent elective coronary angiography through a trans-radial approach at the cardiology department, Zagazig University Hospitals, Zagazig, Egypt.

The patients were divided into two groups. Patient Group: included 100 patients who had radial spasm (32 males and 68 females, with a mean age of 62 ± 5.5 years). The control group included 100 patients who had no radial artery spasm (64 males and 36 females, with a mean age of 53.6 ± 16.9 years). Patients who had acute coronary syndrome or acute heart failure were excluded from the study.

## Methods

All patients were subjected to a complete history taking and a thorough physical examination. Resting 12 lead surface ECG was obtained. Echocardiography was performed using a Hitachi Noblus machine.

Coronary Angiography was performed using Philips Integris 5000, Netherlands. Coronary angiography was done for all patients using the trans-radial approach (right or left). Judkins left or Amplatz left catheters were used for left coronary angiography, and Judkin right, Amplatz right, or 3DRC (William) catheters were used for right coronary angiography provided that all catheters were 6 or 5 French sizes. Local anesthesia of 1.5 ml Xylocaine was administrated 1 inch above the styloid process of radius along the course of the radial artery. The arterial puncture was performed using a 20 Gauge needle. A hydrophilic guide wire was placed into the radial artery and a preloaded ideal hydrophilic sheath (Merit) 6 or 5 French was introduced over it. After insertion of the radial sheath, a cocktail of medication as standard for all patients: 200 µg nitroglycerine and 5000 IU unfractionated heparin. A 100 cm long coronary catheter, preloaded with a 0.035 inch 150 or 260 cm tapered movable core J- wire and in special anatomical loops in the radial artery, we used hydrophilic terumo wire 0.028 260 cm movable core J-wire was advanced through the sheath, and Judkin right, Amplatz right or 3DRC catheters used for right coronary angiography. Then, the catheter was removed and then Judkin left or Amplatz left catheters were used for left coronary angiography. Coronary angiography was performed in multiple projections for adequate analysis of target lesions. If coronary angioplasty was indicated, it was performed in most cases immediately after the diagnostic procedure. After the occurrence of radial artery spasm, if failed to relieve it we shifted to the transfemoral approach to continue the procedure. All those steps were done for the two groups taking into consideration: the access site (right or left) radial artery, number of punctures trials to get the radial artery, size of the radial sheath, number, and size of catheters, procedural time, and presence of radial artery loops. Identification of radial artery spasm which is defined as severe limitation of the catheters movements with or without angiographic confirmation or inability to push or pullback the catheters and if happened we give inside the catheter or the sheath: Another bolus of 200 µg nitroglycerine and 1.25 mg Verapamil.

### Laboratory investigation

Five ml of venous blood was collected for each laboratory investigation under aseptic precautions. Serum was separated immediately for estimation of creatinine and lipid profile levels. Plasma was used for the estimation of blood sugar levels.

### Isolation of DNA

Genomic DNA was extracted from whole blood using a spin column method according to the protocol (QIAamp Blood Kit; Qiagen GmbH, Hilden, Germany). The subjects were genotyped to the Glu298Asp polymorphism (rs1799983) on the eNOS gene by polymerase chain reaction-restriction fragment length polymorphism (PCR-RFLP) as described previously [7]. After digestion with Mbo I enzyme digestion of the 206 bp fragments resulted in fragments that either remained intact (G allele) or were cut into two fragments of 119 bp and 87 bp (T allele). Fragments were analyzed by electrophoresis on 3% agarose gels containing 0.1% ethidium bromide. ( supplementary figure).

### Statistical analysis

The statistical analysis was done with the statistical package SPSS version 20. Categorical variables were presented as percentages. Continuous variables were presented by mean ± SD. Comparisons between groups of means were performed using ANOVA. The genetic equilibrium was tested using Hardy-Weinberg. There were no significant deviations from Hardy-Weinberg equilibrium (P > 0.05), suggesting that our sample was representative of the population. Allele and genotype frequencies in RAS patients and controls were compared using a chi-square test. In addition, the odds ratios (ORs) and 95% confidence intervals (CIs) were calculated as a measure of the association of the eNOS polymorphic sites with RAS. Logistic regression was performed. A value of P < 0.05 was considered statistically significant for all tests.

## Results

Patients of RAS were older than those of the control group (62.04 vs. 53.56, P = 0.000). Females were more common in the RAS group than in the control group (68% vs. 36%, P = 0.000). Regarding risk factors of atherosclerosis e.g. smoking, diabetes, hypertension, dyslipidemia, and family history they were more common in the RAS group compared to the control group (Table 1).

**Table 1:** Demographic data and Risk factors distribution between study groups

	No spasm (n = 100)	Radial spasm (n = 100)	P
Age	53.56 ± 16.9	62.04 ± 5.5	0.000
Female n (%)	36 (36%)	68 (68%)	0.000
Male n (%)	64 (64%)	32 (32%)	0.000
Smoking n (%)	32 (32%)	64 (64%)	0.000
Family history n (%)	16 (16%)	52 (52%)	0.000
Hypertension n (%)	20 (20%)	80 (80%)	0.000
Diabetes mellitus n (%)	32 (32%)	52 (52%)	0.006
Dyslipidemia n (%)	48 (48%)	84 (84%)	0.000
Chronic kidney disease n (%)	16 (16%)	16 (16%)	> 0.05

Regarding drug history, nitrates, acetylsalicylic acid, and insulin were less in the RAS group compared to the control group (Table 2).

**Table 2:** Drug history of the study groups

	No spasm (n = 100)	Radial spasm (n = 100)	P
CCB	16 (16%)	8 (8%)	> 0.05
BB	24 (24%)	24 (24%)	> 0.05
ACEI	52 (52%)	52 (52%)	> 0.05
OHG	8 (8%)	16 (16%)	> 0.05
Insulin	76 (76%)	56 (56%)	0.005
ASA	40 (40%)	16 (16%)	0.000
Nitrates	80 (80%)	44 (44%)	0.000
Statins	28 (28%)	36 (36%)	> 0.05

CCB; calcium channel blockers, BB; beta-blockers, ACEI; angiotensin-converting enzyme inhibitors, OHG; oral hypoglycemic, ASA; acetylsalicylic acid

Regarding the coronary intervention procedure (Table 3): Number of punctures: getting arterial access from the first puncture decreases the incidence of radial spasm. Increasing the attempts to get access increases the risk of radial artery spasm. Radial access: radial artery spasm occurred more with right access in comparison to the left access. Size of radial sheath: radial artery spasm was more liable to present with a 6 F sheath in comparison to the usage of a 5 F sheath during the procedure. The size of catheters: radial artery spasm was more liable to happen with 6 F catheters in comparison to 5 F catheters. Radial tortuosity: the presence of the radial artery loops increases the risk of radial artery spasm (60% vs. 20%) in comparison to the patients who have a normal course of the artery. Procedural time: the longer the procedural time was associated with an increase in the risk of developing radial artery spasm.

**Table 3:** Comparison between the study groups regarding the coronary intervention procedure

		No spasm (n = 100)	Radial spasm (n = 100)	P
Number of punctures	1	64 (64%)	8 (8%)	0.000
	2	16 (16%)	32 (32%)	0.013
	3	20 (20%)	60 (60%)	0.000
Radial Access	Left	92 (92%)	28 (28%)	0.000
	Right	8 (8%)	72 (72%)	0.000
Size of Radial Sheath	5 F	84 (84%)	16 (16%)	0.000
	6 F	16 (16%)	84 (84%)	0.000
Size of Catheters	5 F	84 (84%)	32 (32%)	0.000
	6 F	16 (16%)	68 (68%)	0.000
Number of Catheters	1	8 ( 8%)	4 (4%)	> 0.05
	2	44 (44%)	52 (52%)	> 0.05
	> 3	48 (48%)	44 (44%)	> 0.05
Radial Tortuosity		20 (20%)	60 (60%)	0.000
Procedural time		12.4 ± 2.6	40.9 ± 7.6	0.000

Regarding echocardiography parameters (Table 4): Our results found that LVH increases the incidence of RAS (80% vs. 16%, P < 0.001) and low EF increases the incidence of RAS.

**Table 4:** Comparison between the study groups regarding the Echocardiographic Parameters

	No spasm (n = 100)	Radial spasm (n = 100)	P
WMSI	1.36 ± 0.36	1.46 ± 0.38	0.058
EF	57.4 ± 9.38	54.6 ± 9.82	0.04
Concentric LVH	16 (16%)	80 (80%)	0.000

WMSI; wall motion score index, EF; ejection fraction, LVH; Left ventricular hypertrophy

Genotypes and Alleles distribution between study groups (Table 5): Our results showed that the frequencies of TT genotype of eNOS Glu298Asp gene polymorphism were significantly increased in RAS patients compared to control patients (60% versus 12%). Subjects with the TT genotype and T allele were significantly more likely to develop radial artery spasms (OR = 12.5, 4.6, P < 0.001 respectively).

**Table 5:** Genotypes and Allele distribution in study groups

	No spasm		Radial spasm		OR	CI 95%	P
	N	%	N	%			
*eNOS*							
GG	40	40	16	16			
GT	48	48	24	24	1.25	(0.58–2.7)	> 0.05
TT	12	12	60	60	12.5	(5.4–29.2)	< 0.0001
T allele	72	36	144	72	4.6	(2.99–6.9)	< 0.0001

Our results showed that TT genotype of eNOS gene (Glu298Asp) polymorphism, number of punctures, size of the radial sheath, radial tortuosity, and right radial access are independent predictors of radial artery spasm (Table 6).

**Table 6:** Multivariate Logistic Regression analysis to detect independent predictors of Radial artery spasm Group

Variables	Odds ratio	95% CI	P
Gender	1.538	0.249–9.501	> 0.05
HTN	0.251	0.013–4.865	> 0.05
Dyslipidemia	0.231	0.028–1.912	> 0.05
Concentric LVH	0.131	0.161–60.943	> 0.05
Number of punctures	0.027	0.001–0.508	0.016
RT vs. LT radial	0.013	0.001–0.204	0.002
Size of the radial sheath	21.215	1.823–246.82	0.015
Size of catheter	1.324	0.182–9.655	> 0.05
Radial tortuosity	0.027	0.001–0.490	0.015
eNOS TT Genotype	47.48	3.803–592.840	0.003
eNOS T allele	24.911	1.232–5.432	> 0.05

HTN; hypertension, LVH; left ventricular hypertrophy, eNOS; endothelial nitric oxide synthase

## Discussion

The most important finding of our study is that patients who have TT genotype or T allele of the eNOS gene (Glu298Asp) polymorphism were more likely to develop radial artery spasm during cardiac catheterization (OR = 12.5, 4.6, P < 0.001 respectively). To the best of our knowledge, our study is the first one to demonstrate the association between the eNOS gene (Glu298Asp) polymorphism and the occurrence of RAS during cardiac catheterization.

Previous studies have evaluated coronary artery spasm and eNOS gene polymorphism and concluded that the most important predictive factor for coronary spasm was the Glu298Asp polymorphism of the eNOS gene [11, 12].

Cable et al., [13] found that after adenoviral-mediated eNOS gene transfer to human radial arteries ex vivo, both KCl and prostaglandin F2α-induced contractions were reduced by more than 50% in transduced arteries compared to the controls, suggesting that eNOS gene therapy may exert an important role in the prevention of radial artery graft spasm [14].

Since NO is one of the most important endothelium-derived vasodilators, impaired NO bioavailability plays a crucial role in endothelial dysfunction. eNOS is the predominant isoform of NO synthase and is responsible for the majority of nitric oxide production in the vasculature [15]. The eNOS gene has several single nucleotide polymorphisms reported, one of which is G894T. This polymorphism is a transversion G to T at nucleotide position 894 in exon 7, resulting in a GAG to GAT substitution in exon 7 with the substitution of glutamine by aspartate (Glu298Asp). This variant induces a conformational change that is thought to reduce NOS3 activity and bioavailability [16].

Demographic characteristics and risk factors of patients as predictors of RAS.

Our results showed that older patients were more liable to have RAS than younger ones. This finding supports that of previous studies [17]. This can be explained by aging being associated with endothelial dysfunction [18, 19], oxidative stress in the vasculature is a primary mechanism underlying age-associated reduction in NO bioavailability [19]. Chronic low-grade inflammation has been implicated in age-associated endothelial dysfunction [19]. However, Curtis et al., [20] demonstrated that patients who experienced RAS were younger.

The present study showed that female patients were more liable to have radial artery spasm (68% vs. 32%) in comparison to male patients. This finding goes in concordance with Mercado et al., [10], Jia et al., [21], and Gorgulu et al., [22]. NO production is affected by sex hormones, gender, and age. Estrogen increases eNOS activity [23]. Females are more affected by eNOS Glu298Asp gene polymorphism [24].

Moreover, the radial arteries of women are more sensitive to vasoconstrictors and less sensitive to vasodilators when compared to the radial arteries of men. In addition, women have high sympathetic tone and smaller radial artery lumen which is also a predictor of vasospasm [25]. On the other hand, Tuncez et al., [26] found no significant statistical difference between both gender in developing RAS, this can be explained by a small number of patients who developed RAS in their study, (3 males and 5 females) so there were no sufficient patients to achieve the statistical significance.

The present study showed that hypertensive patients were more liable to develop radial artery spasm (80% vs. 20%) in comparison with the control group (P < 0.001). In agreement with our study, Gorgulu et al., [22] reported that RAS was more common in hypertensive patients (66% vs. 56%, P < 0.009), and Omaygenc et al., [27] found that hypertension was more present in the RAS group compared to no RAS one (78% vs. 61%, P = 0.029).

This can be explained by Hypertension is associated with endothelial dysfunction, accelerated atherosclerosis, accumulation of growth factors and so decrease nitric oxide synthesis which is responsible for maintaining the vasodilatation of the artery [28].

Jia et al., [21] found that hypertension has no effect on developing RAS and this disagreement with our results can be explained by mostly all patients in their study were on ACEIs and ARBs medication and no patients had BB or diuretics. This regimen has a role in improving endothelial dysfunction and NO synthesis.

In our study, we found that diabetic patients were more liable to develop radial artery spasm (52% vs. 32%) in comparison with the control group (P = 0.006).

This can be explained by DM is associated with an increased risk of cardiovascular disease, even in the presence of intensive blood sugar control. Numerous clinical and experimental studies have demonstrated that endothelial dysfunction is closely associated with DM and plays an important role in the progression of atherosclerosis in diabetic patients.

The current study showed that smokers were more liable to develop radial artery spasm (64% vs. 32%, P < 0.001) in comparison with the control group. patients with dyslipidemia were more liable to develop radial artery spasm (84% vs. 48%, P < 0.001) in comparison with the control group.

In concordance with our study, Khan et al., [6] reported that current smokers and patients with dyslipidemia were more liable to develop RAS. Another study demonstrated that the strongest independent predictors of radial occlusion were female sex and active smoking status [29].

This can be explained by smoking as a chronic inflammatory process in which increasing levels of inflammatory markers may induce radial artery spasm. It also increases the sensitivity of alpha-adrenergic receptors which present predominately in the radial artery to the circulating vasoconstrictor materials. Moreover, it has an important role in endothelial dysfunction and hence decreasing the circulating NO which has an important role in maintaining the vasodilatation of the vessels [30].

### Coronary intervention procedure through TRA

The present study showed that radial artery spasm occurs more with ≥ 3 punctures in comparison to just one trial to get access to it, right radial access in comparison to the left access, 6 F sheaths in comparison to 5 F sheaths, 6 F catheters in comparison to 5 F catheters. The presence of radial artery loops increases the risk of radial artery spasm (60% vs. 20%). The procedural time: the longer the procedural time the higher the incidence of RAS. The number of punctures, size of the radial sheath, right radial access, and radial tortuosity are independent predictors of RAS in multivariate analysis.

Those findings in our study can be explained by the radial artery being highly innervated by alpha-adrenergic receptors which highly respond to pain and friction and lead to vasoconstriction so, using larger catheters or sheaths 6 F in comparison to 5 F size will lead to induction of spasm. Also, multiple puncture trials to get access will irritate the vessel with the subsequent release of vasoconstrictor molecules and make the artery more liable to develop spasm. The presence of loops in the radial artery makes the passage of traditional catheters more difficult so, shifting to other catheters and different guide wires with the probability to use a lot of techniques with multiple manipulations to straighten the loops will make the artery liable to develop spasm. Furthermore, the longer the procedural time increases the risk of developing spasm by a lot of manipulation and pain that comes from the movement of the catheters. A previous study showed that pain during radial cannulation and longer procedure time was associated with RAS [31]. Moreover, the right radial artery tends to have more kinks than the left radial artery which leads to excessive manipulation by the catheters during advancement and subsequent spasm [6]. Omaygenc et al., [27] reported that more than one puncture attempt and long procedural time increased with the RAS group compared to no RAS one.

In agreement with our study, Jia et al., [21] found that unsuccessful access from the first attempt, large sheath (6Fa and 7 F) and several catheters > 3, and operation time are independent predictors of RAS. The number of catheters used during the operation was not significant in our study and this disagreement can be explained by using larger catheters (6 or 7 F), and also the female sex was the predominant gender of their cases. In concordance with our study, Gorgulu et al., [22] found that procedural duration and fluoroscopy time were statistically longer in patients with spasm, also, increasing the sheath size increased the spasm incidence and the number of catheters used was not significant. On the other hand, they found that the anatomic variations in radial artery like Loops and hypoplastic artery have no significance in developing spasm and this can be explained by changing the access site and not performing trials to overcome these difficulties to cross by different catheters or wires.

Mercado et al., [10] reported that patients with the following clinical parameters: women over 60 years old, current smokers, previous ischemic heart disease, hypertension, 6Fr catheters, and long procedural time, would have 76% chances of having RAS.

## Conclusion

The eNOS gene polymorphism is associated with RAS during cardiac catheterization in Egyptians. The eNOS (TT) genotype, number of punctures, size of the radial sheath, right radial access, and tortuosity are independent predictors of RAS during cardiac catheterization.

## Electronic supplementary material

Below is the link to the electronic supplementary material.

Supplementary Material 1

## Data Availability

All data and materials are available upon request.

## References

[1] Rashid M, Kwok CS, Pancholy S, Chugh S, Kedev SA, Bernat I, Ratib K, Large A, Fraser D, Nolan J, Mamas MA (2016). Radial artery occlusion after Transradial Interventions: a systematic review and Meta-analysis. J Am Heart Assoc.

[2] Narsinh KH, Mirza MH, Duvvuri M, Caton MT, Baker A, Winkler EA, Higashida RT, Halbach VV, Amans MR, Cooke DL, Hetts SW, Abla AA, Dowd CF (2021). Radial artery access anatomy: considerations for neuroendovascular procedures. J Neurointerv Surg.

[3] Rathore S, Stables RH, Pauriah M, Hakeem A, Mills JD, Palmer ND, Perry RA, Morris JL (2010). Impact of length and hydrophilic coating of the introducer sheath on radial artery spasm during trans-radial coronary intervention: a randomized study. JACC Cardiovasc Interv.

[4] Curtis E, Fernandez R, Lee A (2018). The effect of topical medications on radial artery spasm in patients undergoing transradial coronary procedures: a systematic review. JBI Database System Rev Implement Rep.

[5] Giannopoulos G, Raisakis K, Synetos A, Davlouros P, Hahalis G, Alexopoulos D, Tousoulis D, Lekakis J, Stefanadis C, Cleman MW, Deftereos S (2015) A predictive score of radial artery spasm in patients undergoing transradial percutaneous coronary intervention. Int J Cardiol. 1;188:76–80 10.1016/j.ijcard.2015.04.024 25889332

[6] Khan MZ, Patel K, Franklin S, Faruqi A, Ahmad W, Saeed J (2020). Radial artery spasm: reviews and updates. Ir J Med Sci.

[7] Abdel-Aziz TA, Mohamed RH (2013). Association of endothelial nitric oxide synthase gene polymorphisms with classical risk factors in development of premature coronary artery disease. Mol Biol Rep.

[8] Shahid SU, Shabana, Rehman A (2017). Association patterns of endothelial nitric oxide synthase gene (NOS3) variant Glu298Asp with blood pressure and serum lipid levels in subjects with coronary artery disease from Pakistan. Ann Hum Genet.

[9] Nakayama M, Yasue H, Yoshimura M, Shimasaki Y, Kugiyama K, Ogawa H, Motoyama T, Saito Y, Ogawa Y, Miyamoto Y, Nakao K (1999). T-786–>C mutation in the 5’-flanking region of the endothelial nitric oxide synthase gene is associated with coronary spasm. Circulation.

[10] Mercado N, Rubio M, Cisneros M, Trejo S, Giraudo M (2020). Smoking as an independent predictor of radial spasm. Revista Argentina de Cardioangiología Intervencionista.

[11] Yoshimura M, Yasue H, Nakayama M, Shimasaki Y, Ogawa H, Kugiyama K, Saito Y, Miyamoto Y, Ogawa Y, Kaneshige T, Hiramatsu H, Yoshioka T, Kamitani S, Teraoka H, Nakao K (2000). Genetic risk factors for coronary artery spasm: significance of endothelial nitric oxide synthase gene T-786–>C and missense Glu298Asp variants. J Investig Med.

[12] Chang K, Baek SH, Seung KB, Kim PJ, Ihm SH, Chae JS, Kim JH, Hong SJ, Choi KB (2003). The Glu298Asp polymorphism in the endothelial nitric oxide synthase gene is strongly associated with coronary spasm. Coron Artery Dis.

[13] Cable DG, Caccitolo JA, Pearson PJ, O’Brien T, Mullany CJ, Daly RC, Orszulak TA, Schaff HV (1998). New approaches to prevention and treatment of radial artery graft vasospasm. Circulation.

[14] Chen AF, Ren J, Miao CY (2002). Nitric oxide synthase gene therapy for cardiovascular disease. Jpn J Pharmacol.

[15] Moncada S, Higgs EA (2006). The discovery of nitric oxide and its role in vascular biology. Br J Pharmacol.

[16] Azmy R, Dawood A, Kilany A, El-Ghobashy Y, Ellakwa AF, El-Daly M (2012). Association analysis of genetic variations of eNOS and α2β1 integrin genes with type 2 diabetic retinopathy. Appl Clin Genet.

[17] Gorgulu S, Norgaz T, Karaahmet T, Dagdelen SJJ (2013). Incidence and predictors of radial artery spasm at the beginning of a transradial coronary procedure. J Interv Cardiol.

[18] Celermajer DS, Sorensen KE, Spiegelhalter DJ, Georgakopoulos D, Robinson J, Deanfield JE (1994). Aging is associated with endothelial dysfunction in healthy men years before the age-related decline in women. J Am Coll Cardiol.

[19] Donato AJ, Machin DR, Lesniewski LA (2018) Mechanisms of Dysfunction in the Aging Vasculature and Role in Age-Related Disease. Circ Res. 14;123(7):825 – 48 10.1161/CIRCRESAHA.118.312563 PMC6207260 30355078

[20] Curtis E, Fernandez R, Khoo J, Weaver J, Lee A, Halcomb E (2023). Clinical predictors and management for radial artery spasm: an australian cross sectional study. BMC Cardiovasc Disord.

[21] Jia DA, Zhou YJ, Shi DM, Liu YY, Wang JL, Liu XL, Wang ZJ, Yang SW, Ge HL, Hu B, Yan ZX, Chen Y, Gao F (2010). Incidence and predictors of radial artery spasm during transradial coronary angiography and intervention. Chin Med J (Engl).

[22] Gorgulu S, Norgaz T, Karaahmet T, Dagdelen S (2013). Incidence and predictors of radial artery spasm at the beginning of a transradial coronary procedure. J Interv Cardiol.

[23] Hashimoto M, Miyai N, Hattori S, Iwahara A, Utsumi M, Arita M, Takeshita T (2016). Age and gender differences in the influences of eNOS T-786 C polymorphism on arteriosclerotic parameters in general population in Japan. Environ Health Prev Med.

[24] Periaswamy R, Gurusamy U, Shewade DG, Cherian A, Swaminathan RP, Dutta TK, Jayaraman B, Chandrasekaran A (2008). Gender specific association of endothelial nitric oxide synthase gene (Glu298Asp) polymorphism with essential hypertension in a south indian population. Clin Chim Acta.

[25] Mong K, Duggan JA, Tabrizchi R (2002). Comparative study of functional responses and morphometric state of distal radial arteries in male and female. Ann Thorac Surg.

[26] Tuncez A, Kaya Z, Aras D, Yıldız A, Gül EE, Tekinalp M, Karakaş MF, Kısacık HL (2013). Incidence and predictors of radial artery occlusion associated transradial catheterization. Int J Med Sci.

[27] Omaygenc MO, Karaca IO, Ibisoglu E, Günes HM, Kizilirmak F, Cakal B, Guler E, Barutcu I, Boztosun B (2018). A novel predictor of radial spasm: arterial stiffness. Blood Press Monit.

[28] Nwabuo CC, Vasan RS (2020). Pathophysiology of Hypertensive Heart Disease: Beyond Left Ventricular Hypertrophy. Curr Hypertens Rep.

[29] Schlosser J, Herrmann L, Böhme T, Bürgelin K, Löffelhardt N, Nührenberg T, Mashayekhi CM 1, Neumann FJ, Hochholzer W (2022) Incidence and predictors of radial artery occlusion following transradial coronary angiography: the proRadial trial. Clinical Research in Cardiology 10.1007/s00392-022-02094-z PMC10449957 36074269

[30] Sugiishi M, Takatsu F (1993). Cigarette smoking is a major risk factor for coronary spasm. Circulation.

[31] Ruiz-Salmerón RJ, Mora R, Vélez-Gimón M, Ortiz J, Fernández C, Vidal B, Masotti M, Betriu A (2005). Radial artery spasm in transradial cardiac catheterization. Assessment of factors related to its occurrence, and of its consequences during follow-up. Rev Esp Cardiol.

