# Supplementary figures and images for: Association of endothelial nitric oxide synthase (Glu298Asp) gene polymorphism with radial artery spasm during cardiac catheterization in Egyptians
Source: Mol Biol Rep. 2023 May 23;50(7):5747–53. doi: 10.1007/s11033-023-08434-0 (PMC10289915; doi:10.1007/s11033-023-08434-0)

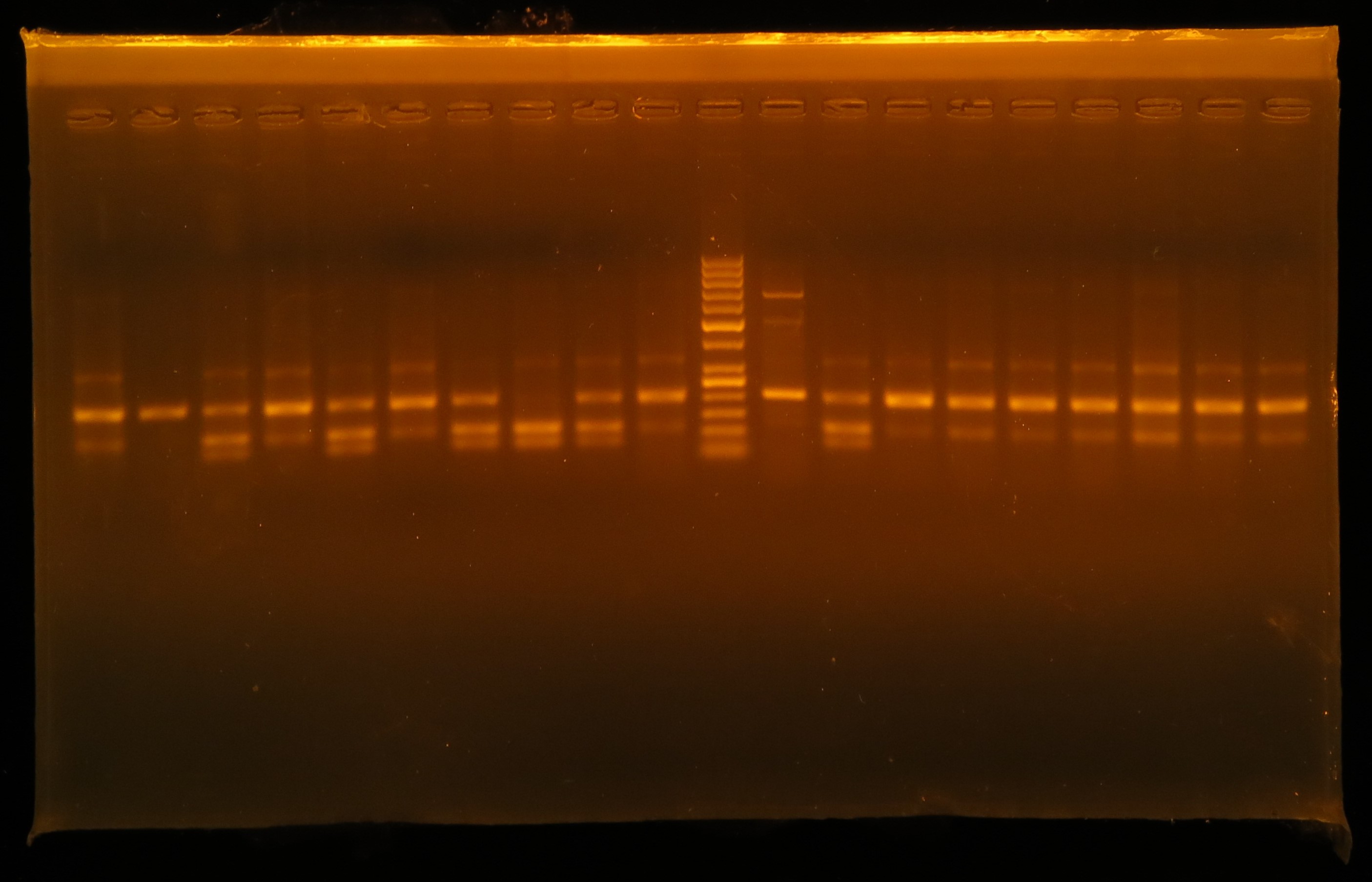

Supplement: Supplementary file 1 — Supplementary Material 1 [file 11033_2023_8434_MOESM1_ESM.jpg]
